# Combination treatment with hypofractionated radiotherapy plus IL-2/anti-IL-2 complexes and its theranostic evaluation

**DOI:** 10.1186/s40425-019-0537-9

**Published:** 2019-02-26

**Authors:** Hua Jing, Michael Hettich, Simone Gaedicke, Elke Firat, Mark Bartholomä, Gabriele Niedermann

**Affiliations:** 1 Department of Radiation Oncology, Medical Center, Faculty of Medicine, University of Freiburg, Freiburg, Germany; 2 Department of Nuclear Medicine, Medical Center, Faculty of Medicine, University of Freiburg, Freiburg, Germany; 3 German Cancer Consortium (DKTK) Partner Site Freiburg, Freiburg, Germany; 4 German Cancer Research Center (DKFZ) , Heidelberg, Germany; 5 Innovent Biologics (Suzhou) Co., Ltd., Suzhou, China; 6 Oncology Translational Imaging Science, Roche pRED, Basel, Switzerland

**Keywords:** Cancer immunotherapy, Radiotherapy, IL-2, Cytokine (complex) therapy, PET imaging

## Abstract

**Background:**

Immunogenic radiotherapy (RT) can act synergistically with immune checkpoint blockers (ICBs). However, alternatives are needed for non-responding patients and those with pre-existing or ICB-induced autoimmune symptoms. Combination of RT with IL-2 could be an alternative. But IL-2 has a short half-life, and, by binding to its high-affinity receptor, it strongly stimulates immunosuppressive CD4+ Tregs and seems to promote potentially life-threatening vascular leakage. IL-2/anti-IL-2 complexes (IL-2c), which bind to the low-affinity receptor, have been reported to circumvent these disadvantages but they have not yet been thoroughly tested in conjunction with radiotherapy.

**Methods:**

We evaluated, in three mouse models, the antitumoral effects induced by hypofractionated RT (hRT) plus IL-2c. We also used non-invasive imaging with a newly developed PET tracer based on therapeutically active IL-2c and a PD-L1 PET tracer for the theranostic evaluation of the treatment and its side effects.

**Results:**

Treatment of mice bearing established B16 melanomas with hRT + IL-2c was superior to hRT + uncomplexed IL-2 or hRT alone; IL-2c alone was not effective. hRT + IL-2c was also synergistic in mice bearing C51 colon carcinomas or 4T1 mammary carcinomas. The better antitumor response correlated with increased tumor-specific CD8+ T cells and NK cells, but not CD4+ Tregs, in the irradiated tumor and in lymphoid organs. With the new PET tracer, we visualized the whole-body distribution of IL-2c and the bound receptors in naïve mice and tumor-bearing mice. Surprisingly, the tumor uptake was non-specific and only moderate. This prompted experiments demonstrating that specific IL-2c binding in the tumor is limited by IL-2 secreted by tumor-resident effector cells and that extratumorally expanded T and NK cells can infiltrate the irradiated tumor, which suggests that systemic immune activation considerably contributed to the reduction of tumor growth. Lastly, we show that a side effect of IL-2c treatment – a quite dramatic non-specific expansion of CD8+ T and NK cells – is only transient, and we visualized the associated splenomegaly as well as side effects on liver and lung by contrast-enhanced CT and PD-L1 PET.

**Conclusions:**

Our results show that the combination of immunogenic RT with IL-2c that are directed towards the low-affinity IL-2 receptor can be synergistic and more effective than the combination with uncomplexed IL-2. In addition, our theranostic evaluation provided insights into the mechanism of action and the side effects of IL-2c treatment.

**Electronic supplementary material:**

The online version of this article (10.1186/s40425-019-0537-9) contains supplementary material, which is available to authorized users.

## Background

The cytokine IL-2 is mainly secreted by effector T cells and is crucial for the proliferation, differentiation, and survival of T cells, including immunosuppressive CD4+ FoxP3+ regulatory T cells (Tregs). It is also important for natural killer (NK) cells [1–3]. IL-2 has a low molecular mass (15.5 kDa), resulting in a short half-life in the circulation [4]. The high-affinity IL-2 receptor, which is composed of the alpha (CD25) and beta (CD122) chains and the common gamma chain (γ_c_, CD132), is transiently expressed on recently activated T cells and constitutively on CD4+ Tregs. IL-2 binds with lower affinity to the dimeric receptor consisting of CD122 and γ_c_, which is expressed at high levels on memory CD8+ T cells and NK cells [3, 5].

IL-2 exhibits antitumor effects in a small subpopulation of patients with antigenic tumor types such as malignant melanoma or renal cell cancer [4]. Besides the limited therapeutic efficacy, one major disadvantage, particularly of high-dose IL-2, is the induction of the capillary leak syndrome [6], a potentially life-threatening adverse effect. Although the pathophysiology of the IL-2-induced capillary leak syndrome seems multifactorial, binding of IL-2 to CD25 expressed on vascular endothelial cells has been reported to promote it [7, 8].

**Fig. 1:**
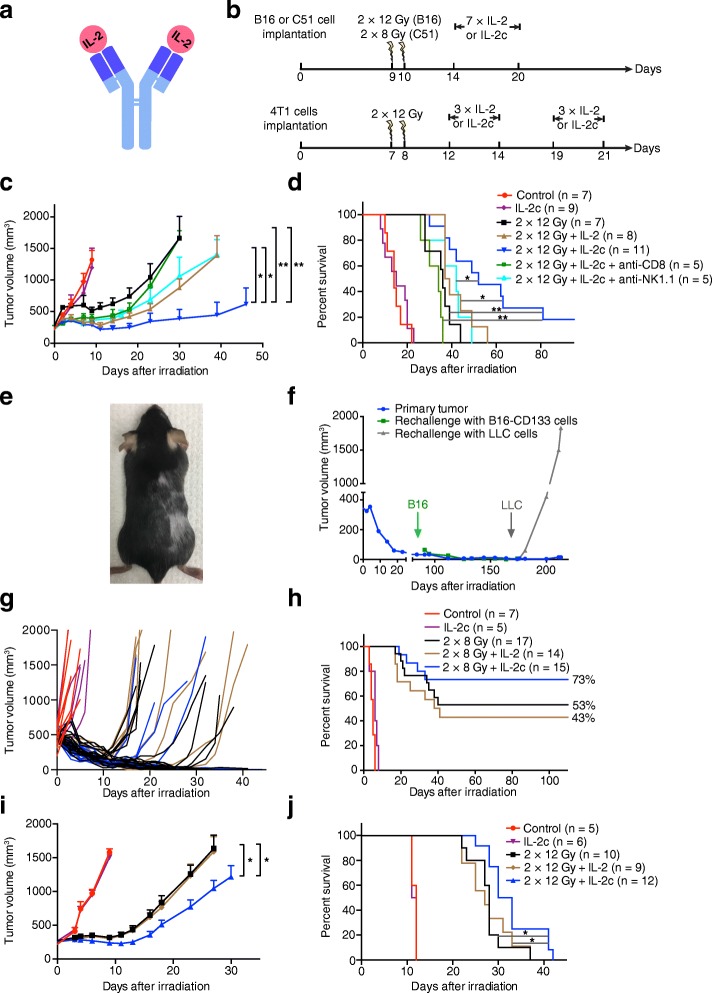
Better tumor control and overall survival of tumor-bearing mice after combination treatment with hRT and IL-2c. **a** Scheme of IL-2c. **b** Treatment scheme. In the combination treatment groups, the daily i.p. injections of equimolar doses of IL-2 or IL-2c started 4 days after hRT. Tumor growth (**c**) and survival (**d**) of CD133-B16 melanoma-bearing mice which were either untreated or treated with IL-2c, 2 × 12 Gy, 2 × 12 Gy + IL-2, or 2 × 12 Gy + IL-2c. The effects of the depletion of either CD8+ T cells or NK cells are also shown. **e** Long-term survival of mice after combined treatment with hRT and IL-2c is associated with the development of vitiligo. **f** Mice cured from established B16 tumors are immune to re-challenge with CD133-B16 cells but not LLC cells. **g**, **h** Tumor growth (**g**) and survival (**h**) of mice bearing C51 colon carcinomas which were either untreated or treated with IL-2c, 2 × 8 Gy, 2 × 8 Gy + IL-2, or 2 × 8 Gy + IL-2c. **i**, **j** Tumor growth (**i**) and survival (**j**) of mice bearing 4T1 mammary carcinomas which were either untreated or treated with IL-2c, 2 × 12 Gy, 2 × 12 Gy + IL-2, or 2 × 12 Gy + IL-2c

Various strategies have been employed to enhance the therapeutic efficacy and reduce the adverse effects of IL-2. These include tumor-targeted variants; another strategy is to avoid the binding of IL-2 to CD25 either by mutation or by complex formation with anti-IL-2 antibodies such as the S4B6 monoclonal antibody [1, 5, 9, 10] (Fig. 1a). CD122/γ_c_-directed IL-2/anti-IL-2 complexes (IL-2c) do not bind CD25 [11–13] and increase the circulation time of IL-2 [11, 13, 14]. They therefore strongly expand cytotoxic T (CD8+) and NK cells but cause only little expansion of immunosuppressive Tregs [13, 15, 16]. In addition, treatment with IL-2c has been reported to reduce the capillary leak syndrome [7, 8].

The therapeutic efficacy of IL-2 can also be enhanced by combination treatments, e.g., with local radiotherapy (RT). Suggested by early studies in mice [17], this could not be reproduced in a subsequent clinical trial published in 1992 [18]. However, a recent trial suggested strong local and systemic antitumor activity of combinations of high-dose radiation and IL-2 in metastatic melanoma and renal cell carcinoma patients [19]. Both trials utilized hypofractionated RT (hRT), which is delivered in fewer fractions than conventional RT, but in the early trial, the dose per fraction and the biologically effective RT dose were comparatively low. In recent years, it has repeatedly been shown that hRT with moderate or higher-dosed fractions can induce tumor-specific CD8+ cytotoxic T cells [20–22]. A major contributing factor is the induction of immunogenic cell death [23]. In addition, tumor irradiation enhances the influx of T cells into tumors. Accordingly, combinations of tumor immunotherapeutics such as immune checkpoint blockers (ICBs), IL-2, IL-2 variants, or others, with immunogenic RT can be synergistic [17, 19, 20, 22, 24–27].

The combination of RT and CD122/γ_c_-directed IL-2c has recently been studied in a two-tumor sarcoma mouse model where only one of the two tumors was irradiated. In this highly radiosensitive model, control of the irradiated tumor and CD8+ tumor-infiltrating lymphocytes (TILs) did not differ between the RT/IL-2c combination and RT monotherapy groups, and the control of unirradiated tumors did not differ between the RT/IL-2c combination and IL-2c monotherapy groups [28]. Here, we investigated the antitumor efficacy of immunogenic hRT + CD122/γ_c_-directed IL-2c in models of aggressively growing B16 melanoma as well as C51 colon and 4T1 mammary carcinomas and found synergistic effects of immunogenic RT and IL-2c. In addition, we developed a PET tracer that allows the non-invasive visualization of the biodistribution of CD122/γ_c_-directed IL-2c and their binding to the low-affinity IL-2 receptor (CD122/γ_c_). The PET imaging and biodistribution experiments helped to reveal that CD122/γ_c_-directed IL-2c preferentially bind CD122+ cells outside of the tumor and that target binding in the tumor can be limited by IL-2 secreted by tumor-resident effector cells. We also used non-invasive imaging with PET and contrast-enhanced CT to visualize the transient splenomegaly and other side effects caused by IL-2c-mediated unspecific bystander T and NK cell activation.

## Methods

### Cell lines

The B16F10 melanoma cell line was obtained from H.P. Pircher (Freiburg) and was authenticated by shape/morphology, intracellular TRP-I staining, and recognition by *pmel-1* transgenic T cells. Cells were transduced with lentiviral particles encoding the human stem cell marker CD133, as described before [29], and sorted for CD133 expression. The Lewis lung carcinoma cell line LLC-1 (CRL-1642) and the 4T1 mammary carcinoma cell line were purchased from ATCC. The C51 colon carcinoma cell line was obtained from Mario Paolo Colombo (Milan) [30].

### Mice

All animal experiments were performed in accordance with the German Animal License Regulations and were approved by the animal care committee of the Regierungspräsidium Freiburg (registration number: G-13/082). C57BL/6 N and BALB/c mice were purchased from Janvier Labs and kept under standard pathogen-free conditions.

### Tumor models

Tumor cells (2 × 10^5^) dissolved in 50% matrigel were implanted into the right flank of 8–12-week-old C57BL/6 N mice (CD133-expressing B16F10 cells) or BALB/c mice (C51 or 4T1 cells). The growth of the xenografts was monitored by caliper measurements. The tumor volume was calculated using the formula: length × width × height. When the tumors reached 250 mm^3^ (radioresistant B16 and 4T1 models) or 500 mm^3^ (radiosensitive C51 model), the mice were randomized before treatment. Tumors were irradiated locally with two fractions of 12 Gy (2 × 12 Gy; B16 and 4T1 models) or 8 Gy (2 × 8 Gy; C51 model) on consecutive days as described previously [31], followed by daily intraperitoneal (i.p.) injections of equimolar amounts of IL-2 (1.5 μg, corresponding to 7500 IU, PeproTech) or IL-2c (9 μg, corresponding to 7500 IU IL-2) on days 5–11 (B16 and C51 models) or on days 5–7 and 12–14 (4T1 model) (Fig. 1b). Different tumor sizes and RT fraction doses were chosen because of differences in radiosensitivity between the tumor models (see above). In the poorly immunogenic 4T1 model, we thought that extending the time period of IL-2c dose administration might increase treatment efficacy. Mice were sacrificed when their tumors reached a size of 2000 mm^3^. In the highly metastatic 4T1 model, some mice had to be sacrificed because of lung metastases (as evidenced by loss of body weight and low activity and as confirmed by necropsy) before the subcutaneous (s.c.) tumor reached 2000 mm^3^. Re-challenge experiments in cured mice were performed by injecting 2 × 10^4^ tumor cells dissolved in 50% matrigel.

### In vivo depletion of CD8+ T cells and NK cells

CD8+ cell-depleting antibodies (clone 2.43, BioXcell, 200 μg) or NK cell-depleting antibodies (clone PK136, BioXCell, 100 μg) were injected i.p. 5 days and 1 day before treatment and thereafter once a week. CD8+ T cell and NK cell depletion was confirmed by flow-cytometrical analysis of peripheral blood.

### Adoptive transfer of extratumoral T and NK cells

To obtain CD8+ T cells and NK cells for adoptive transfer experiments, B16-CD133 tumor-bearing mice were locally irradiated with 2 × 12 Gy and treated thereafter with IL-2c as described above. One day after the fifth IL-2c injection, CD8+ T cells and NK cells were isolated from spleen, lymph nodes, and blood using kits from Miltenyi Biotech. Thereafter, the isolated cells were labeled with 5 μM Cell Trace Violet (Invitrogen) according to the manufacturer’s instructions. Recipient mice were also treated as described above, and, without prior lymphodepletion, 6 × 10^6^ CD8+ or 9 × 10^6^ NK1.1+ cells labeled with Cell Trace Violet were injected intravenously (i.v.) into recipient mice 3 days after the second irradiation; at the same day, daily IL-2c treatment was started. At day 3 after the transfer, the proportions of Cell Trace Violet+/CD8+ cells, Cell Trace Violet+/NK1.1+ cells and of M8-Tetramer+/CD8+/Cell Trace Violet+ cells in spleen and tumor were determined by flow cytometry. In addition, cell divisions of the transferred T and NK cells were analyzed by flow-cytometric determination of Cell Trace Violet dilution.

### Preparation of IL-2c

S4B6 anti-mIL-2 mAb [32] was purified from culture supernatants of the S4B6–1 hybridoma (ATCC® HB-10968™) according to standard methods. IL-2c were generated by incubating recombinant murine IL-2 with S4B6 anti-mIL-2 mAb at a 2:1 M ratio at 37 °C for 30 min, before i.p. injection into animals. The formation of IL-2c was examined by performing size exclusion chromatography on an Enrich™ SEC 650 column (Bio-Rad).

### Preparation of radiolabeled antibodies

S4B6 anti-IL-2 mAb or anti-PD-L1 (10F.9G2) mAb were conjugated with the metal chelator *S*-2-(4-isothiocyanatobenzyl)-1,4,7-triazacyclononane-1,4,7-triacetic acid (*p*-SCN-Bn-NOTA) as described before [29]. IL-2c were generated after the labeling of NOTA-S4B6 mAb with radioactive ^64^Cu as described above. In vivo IL-2c binding specificity was tested with the same mAb labeled with CF680 (CF680 VivoBrite Rapid Antibody Labeling Kits for Small Animal In Vivo Imaging, Biotium) according to the manufacturer’s instructions.

### ImmunoPET and CT imaging

ImmunoPET was performed 24 h after i.v. injection of either 5 μg ^64^Cu-NOTA-IL-2c or 20 μg ^64^Cu-NOTA-PD-L1 mAb using a microPET Focus 120 (Concorde), immediately followed by CT imaging in two bed positions with a tube voltage of 40 keV and a tube current of 1 mA (microCT scanner; CT Imaging). For contrast-enhanced CT imaging of spleens, 30 mg of ExiTron nano 12,000 (Miltenyi Biotec) was injected i.v. into mice, immediately followed by CT imaging. Mice were anesthetized using 2% isoflurane/O_2_ during PET/CT imaging. The scanning duration for immunoPET was dependent on the injected activity and the elapsed time after tracer injection. ImmunoPET scans were acquired for 15–20 min. For all scans, a total count of at least 4.5 million was recorded. To deplete CD122+ cells, 250 μg anti-CD122 antibody (eBioscience) was injected i.p. 24 h prior to the injection of the ^64^Cu-NOTA-IL-2c tracer.

### PET/CT image analysis

A routine 2D ordered subset expectation maximization (OSEM2D) algorithm provided by the scanner software was used to reconstruct PET images with a resolution of 1.5 mm. CT scans were reconstructed with a resolution of 120 μm and a T30 kernel, using the software provided by the manufacturer. Fusion of the PET and CT images was performed with AMIDE software 1.0.5 [33].

### Ex vivo biodistribution

After PET/CT imaging, mice were euthanized, organs and blood were collected, flushed and weighed, and the activity was measured with a Wizard [2] gamma counter (PerkinElmer). All values were decay and background corrected and expressed as percentage of the injected activity per gram tissue (% IA/g) using a standard of 100% of the injected dose. Left- and right-side lymph nodes were pooled for biodistribution analysis.

### Flow cytometry

Flow-cytometric analysis of single-cell suspensions from spleen, lymph nodes (inguinal, axillary, cervical and mesenteric lymph nodes), thymus, whole blood, bone marrow, and TILs was performed using α-CD45, α-CD8, α-CD4, α-CD19, α-CD44, α-CD25, α-CD122, α-FoxP3, α-IFN-γ (all eBioscience), α-TNF-α, α-Ki67, α-CD3 (BioLegend), α-NK1.1 (BD Pharmingen). Stainings for intracellular FoxP3, Ki67, TNF-α, and IFN-γ were performed following the manufacturer’s instructions (eBioscience).

### Determination of tumor-specific T cells

Before intracellular IFN-γ and TNF-α staining, the cells were incubated either with IFN-γ-stimulated (50 IU/ml; 48 h), 40-Gy-irradiated CD133-positive B16 cells or with gp-70 peptide (C51 tumor model) for 6 h at 37 °C in the presence of brefeldin A (eBioscience). PE-labeled H-2K^b^-restricted MuLV p15E_604–611_ tetramer was purchased from Baylor College of Medicine. Stained cells were analyzed using a BD FACSVerse flow cytometer with FACSuite software (Becton Dickinson).

### IL-2/IL-2c competition experiment

Isolated CD8+ T cells were preincubated with different concentrations of IL-2 or interferon-β (R + D Systems) for 30 min at 4 °C. Then, 1 μg IL-2c was added and the cells were further incubated for 30 min at 4 °C. For detection of the bound IL-2c, the washed cells were incubated with a PE-labeled anti-rat IgG2a secondary antibody (recognizing the S4B6 antibody) at a concentration of 0.25 μg per sample for 30 min at 4 °C. Thereafter, the cells were incubated with CD8-PE-Cy7 and CD122-APC antibodies and analyzed by FACS.

### Statistical analysis

Results are presented as mean ± SEM. Statistical analyses were carried out using GraphPad Prism 6.0 statistical software (GraphPad Software Inc.). Survival curves were compared using the log-rank test. A *p*-value < 0.05 was considered significant (*p < 0.05, **p < 0.01, ***p < 0.001, and ****p < 0.0001).

## Results

### Synergistic antitumor effects of hRT and IL-2c in mouse models with established tumors

Initially, we tested the antitumor efficacy of hRT + IL-2c in mice bearing radioresistant but antigenic B16 melanoma tumors expressing human CD133 as a foreign antigen. When the tumors had reached approximately 250 mm^3^, mice were treated with IL-2c alone, hRT alone, hRT + IL-2, or hRT + IL-2c (Fig. 1b). While IL-2c alone did not inhibit the tumor growth and hRT alone delayed it only for a short period of time, the hRT/IL-2c combination was much more effective. Moreover, tumor growth suppression by hRT + IL-2c was significantly better than that by hRT + IL-2 (Fig. 1c). This was also reflected by the overall survival, which was best for the hRT + IL-2c group. Moreover, complete cures were only observed in this group (Fig. 1d). The cured mice showed pronounced vitiligo at and around the site of irradiation, indicating the induction of cross-reactive T cells with shared specificity against melanoma and melanocyte antigens (Fig. 1e). The induction of tumor antigen-specific memory T cells was suggested by the observed protection of cured mice against re-challenge with B16 melanoma cells but not with Lewis lung carcinoma cells (LLC-1) (Fig. 1f). A crucial role of CD8+ T cells for tumor reduction and survival upon hRT + IL-2c was proven by antibody-mediated CD8+ T cell depletion. The depletion of NK cells did also negatively affect tumor growth control and survival, albeit to a lesser extent (Fig. 1c,d).

We confirmed the enhanced treatment efficacy of hRT + IL-2c in mice bearing s.c. C51 colon carcinomas (Fig. 1b, g, and h). C51 colon carcinomas are quite sensitive to radiation treatment per se, resulting in a cure rate of 53% even when only two fractions of 8 Gy were administered on two consecutive days. However, the cure rate rose to 73% when the mice were treated with hRT + IL-2c. In contrast, the cure rate after treatment with hRT + free IL-2 was similar to that after hRT alone. Except for one mouse, all cured mice re-challenged with C51 tumor cells 2 months after complete tumor regression were protected against the re-challenge, independent of whether they initially received IL-2c in addition to hRT or only curative hRT (data not shown).

In mice bearing radioresistant and poorly immunogenic s.c. 4T1 tumors, we also observed a synergistic effect of hRT and IL-2c (Fig. 1i and j).

### Increases in intra- and extratumoral CD8+ T cells and NK cells, including tumor-specific T cells

**Fig. 2:**
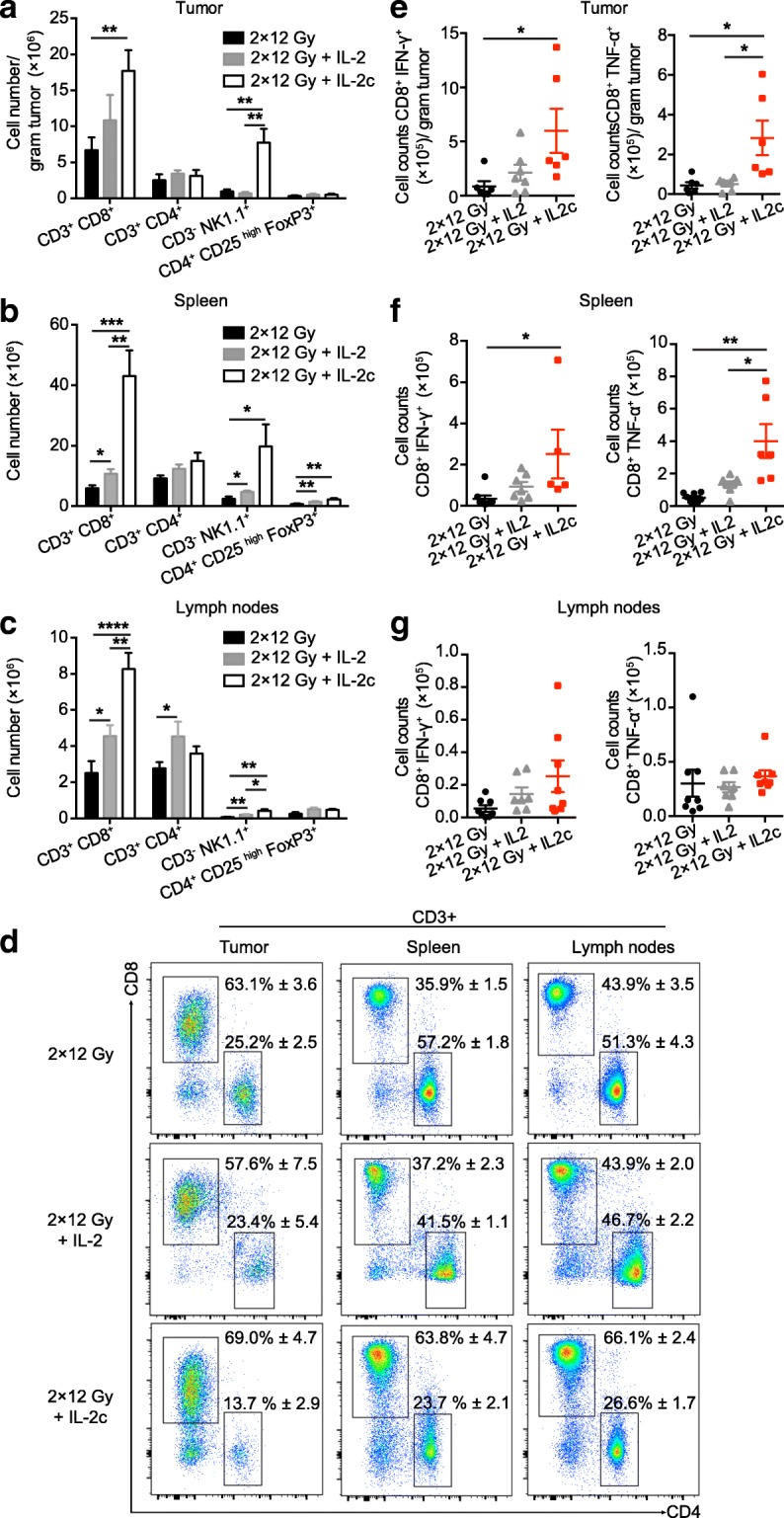
hRT**/**IL-2c combination treatment enhances the numbers of tumor-specific CD8+ T cells in the CD133-B16 melanoma model. Mice bearing an s.c. B16 melanoma tumor were treated with hRT alone or hRT followed by daily i.p. injections of IL-2 or IL-2c for five consecutive days. One day later, tumor (**a**), spleen (**b**), and lymph nodes (**c**) were collected and analyzed by flow cytometry for total counts of the indicated cell subsets (*n* ≥ 7). **d** Examples of the flow cytometry results showing proportions of CD8+ and CD4+ T cells in tumor, spleen, and lymph nodes, after hRT, hRT + IL-2, or hRT + IL-2c. **e**-**g** Quantification of IFN-γ- and TNF-α-producing tumor-specific CD8+ cells from tumor, spleen, and lymph nodes after restimulation on IFN-γ-pretreated, irradiated B16-CD133 melanoma cells (*n* ≥ 4 mice/group)

The superior antitumor response to hRT + IL-2c compared to hRT monotherapy correlated with a strong increase in the numbers of CD8+ T cells and NK cells in the tumor as well as in secondary lymphoid organs (Fig. 2a-c). In the melanoma model, CD8+ T cell numbers per gram tumor increased from (6.7 ± 1.8) × 10^6^ (hRT) to (1.8 ± 0.3) × 10^7^ (hRT + IL-2c) (Fig. 2a). In contrast, CD4+ T cells and the subpopulation of immunosuppressive CD4+ Tregs increased only slightly in secondary lymphoid organs and the irradiated tumor. The preferential expansion of CD8+ T cells compared to CD4+ T cells is also demonstrated in the flow cytometry plots shown in Fig. 2d. Importantly, the numbers of effector cytokine-producing, tumor-specific CD8+ T cells also increased both in the tumor and in secondary lymphoid organs (Fig. 2e-g). Consistent with the tumor growth and survival data, T and NK cell numbers in mice treated with hRT + free IL-2 were generally more similar to those after hRT monotherapy (Fig. 2a-g).

**Fig. 3:**
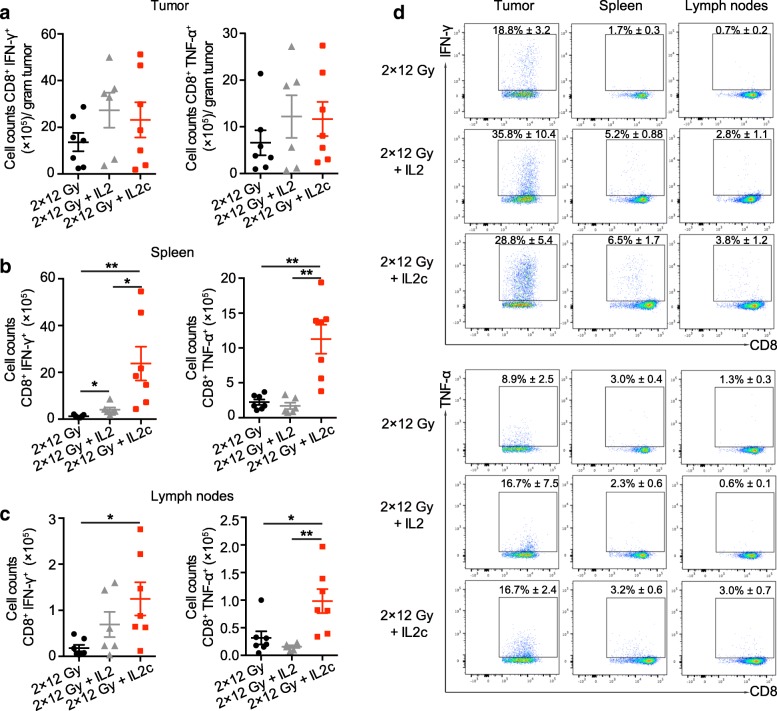
hRT**/**IL-2c combination treatment dramatically enhances tumor-specific CD8+ T cells in secondary lymphatic organs in the C51 colon carcinoma model. Mice bearing an s.c. C51 colon carcinoma tumor were treated with hRT alone or hRT followed by daily i.p. injections of IL-2 or IL-2c for five consecutive days. One day later, IFN-γ- and TNF-α-producing tumor-specific CD8+ cells from tumor (**a**), spleen (**b**), and lymph nodes (**c**) were quantified after restimulation on gp70 peptide-pulsed cells. **d** Flow cytometry results showing proportions of IFN-γ + CD8+ T cells and TNF-α + CD8+ T cells from tumor, spleen, and lymph nodes

In the C51 colon carcinoma model, changes in total numbers of CD8+ T cells, NK cells, and immunosuppressive CD4+ Tregs in spleen and lymph nodes were similar to those in the B16 melanoma model (data not shown). In this highly radiosensitive model, only slight differences were found for tumor-specific CD8+ TILs per gram tumor tissue between the hRT monotherapy, hRT/IL-2 and hRT/IL-2c combination groups (Fig. 3a). However, in hRT/IL-2c-treated mice, we observed a strong increase in the absolute numbers of tumor-specific CD8+ T cells in secondary lymphoid organs (Fig. 3b and c). Note that the number of IFN-γ-producing tumor-specific CD8+ T cells in the spleen increased from (1.2 ± 0.2) × 10^5^ (hRT) to (2.4 ± 0.7) × 10^6^ (hRT + IL-2c). There was also a trend towards a greater proportion of tumor-specific T cells within the CD8+ population in the compartments analyzed (Fig. 3d). Differences in the CD4/CD8 ratio or in the proportion of tumor-specific T cells between tumor-draining and non-draining lymph nodes were not observed (data not shown).

### Non-invasive visualization of IL-2c and of bound receptors in naïve mice

**Fig. 4:**
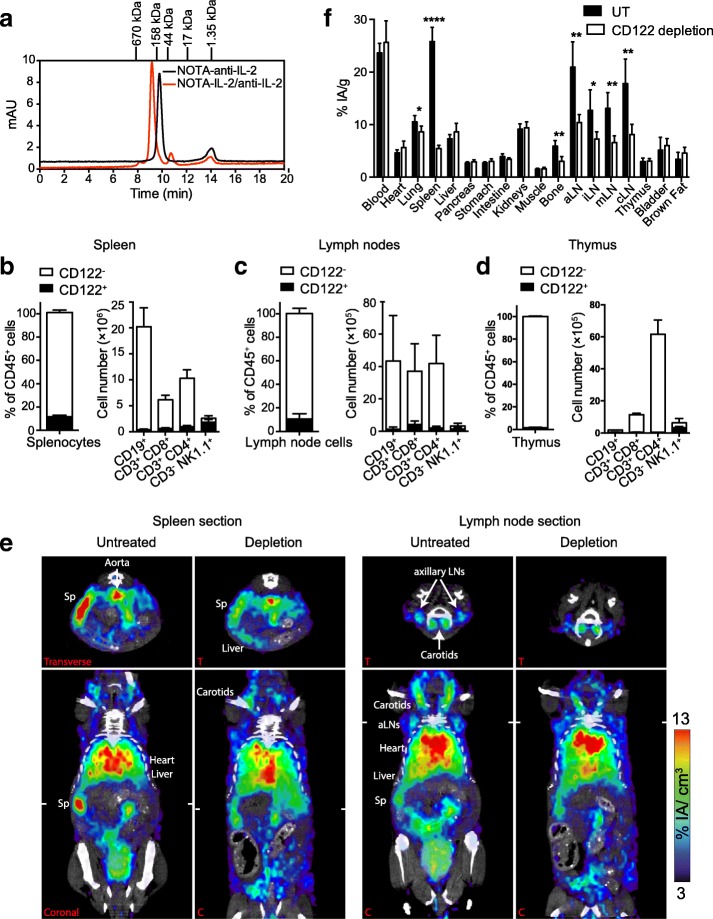
Non-invasive visualization of IL-2c and of bound receptors by immunoPET/CT with ^64^Cu-NOTA-IL-2c in naïve mice. **a** Size exclusion chromatography of ^64^Cu-NOTA-IL-2c, demonstrating complex formation of the S4B6 anti-IL-2 mAb with IL-2; note the shift in the retention time compared to the uncomplexed S4B6 anti-IL-2 mAb; NOTA-conjugated anti-IL-2 mAb was incubated with IL-2 at a ratio of 1:2 at 37 °C for 30 min. 5 μg each of the ^64^Cu-labeled NOTA-conjugated anti-IL-2 mAb and of IL-2c were run on an Enrich™ SEC 650 column. Frequency of CD122+ cells in spleen (**b**), lymph nodes (**c**), and thymus (**d**) of naïve mice (*n* = 3). **e** Representative coronal (C) and transverse (T) immunoPET/CT sections of untreated and CD122-depleted mice 24 h after injection of ^64^Cu-NOTA-IL-2c (5 μg, 7.5 MBq). CD122-depleted mice were pre-treated with 0.25 mg depletion mAb (i.p.) 24 h before tracer injection. White ticks in the C-sections indicate the positions of the T-sections. The scanning duration was approximately 15–20 min. (f) Ex vivo biodistribution of radiotracer uptake 24 h after injection; *n* = 4 mice per group. Abbreviations: cLN – cervical lymph node, aLN – axillary lymph node, iLN – inguinal lymph node, mLN – mesenteric lymph node, Sp – spleen

To visualize the biodistribution of IL-2c and the bound low-affinity (CD122/γ_c_) receptors, we developed a novel PET tracer. For this purpose, S4B6 (anti-IL-2) antibodies were conjugated with the chelator NOTA and, shortly before the PET experiment, the conjugates were loaded with ^64^Cu and then complexed with IL-2. The successful complex formation with IL-2 was proven by a characteristic shift in the retention time in size exclusion chromatography (Fig. 4a).

Under physiological conditions, CD122 is mainly expressed on subpopulations of T cells and on NK cells [1]. Accordingly, in naïve, healthy mice, we detected CD122 only on 11.7 ± 0.8% and 10.7 ± 2.5% of cells in spleen and lymph nodes, respectively (Fig. 4b and c). The frequency of CD122+ cells in the thymus, a primary lymphoid organ, was considerably lower (less than 2%; Fig. 4d).

Despite the low expression of CD122, we successfully imaged the whole-body distribution of the IL-2c and the bound CD122/γ_c_ receptors in naïve mice. As shown in Fig. 4e, microPET imaging with ^64^Cu-NOTA-IL-2c enabled visualization of the spleen and at least some lymph nodes 24 h post injection (p.i.). To confirm the immunoPET specificity, we depleted CD122-positive cells with a depleting antibody (Additional file 1), which was injected 24 h before tracer injection. As shown in Fig. 4e and Additional file 2, the spleen and the lymph node signals were strongly reduced in mice in which CD122+ cells were depleted. These observations were confirmed by ex vivo biodistribution analysis 24 h p.i., where specific tracer uptake was found in the spleen, lymph nodes, and bones, and to a minor extent in the lung, but not in the thymus (Fig. 4f).

### IL-2c immunoPET in tumor-bearing mice

**Fig. 5:**
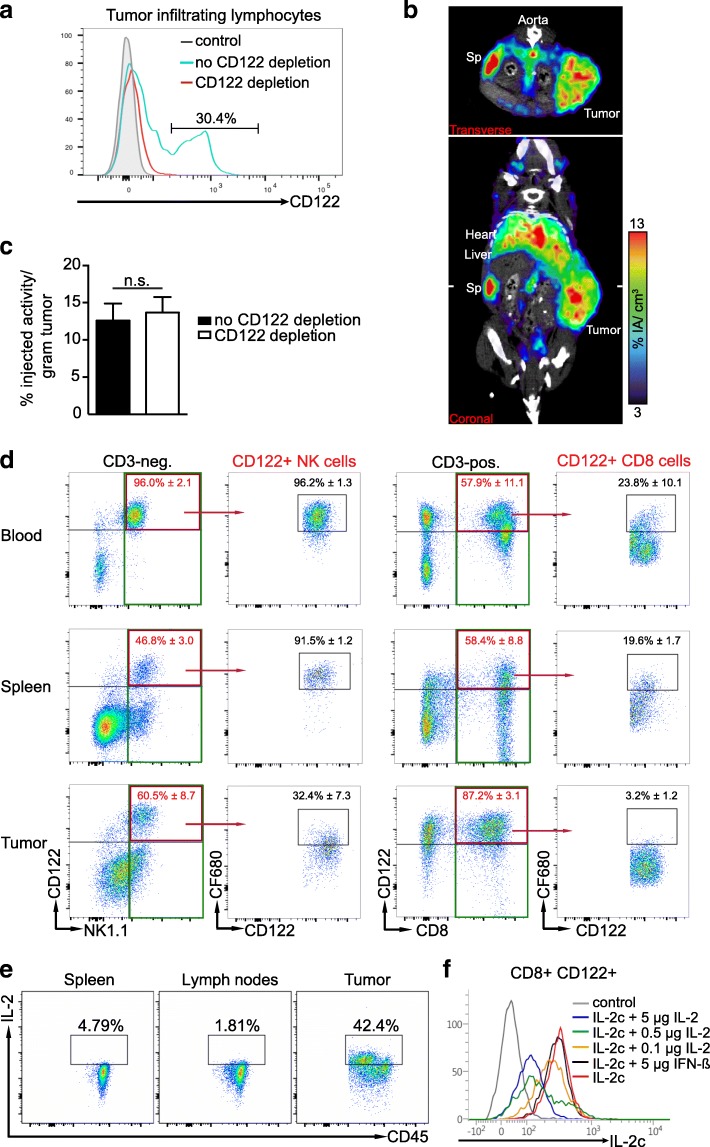
IL-2c immunoPET/CT in tumor-bearing mice and analyses of IL-2c target binding in blood, spleen, and tumor. **a** Flow cytometry analysis of CD122+ tumor-resident leukocytes 5 days after hRT in mice with or without depletion of CD122+ cells. **b**, **c** Mice bearing an s.c. B16 tumor were locally irradiated with 2 × 12 Gy when their volume reached 400 mm^3^. Five days later, they received 5 μg ^64^Cu-NOTA-IL-2c (~ 7.5 MBq) and PET images were acquired 24 h later. **b** Representative coronal and transverse IL-2c immunoPET/CT sections are shown. White ticks in the C-sections indicate the positions of the T-sections. **c** Ex vivo biodistribution of radiotracer uptake 24 h p.i. in irradiated tumors of CD122-depleted and non-depleted mice. **d** Binding of IL-2c-CF680 with CD122+ NK and CD8+ T cells in vivo. IL-2c-CF680 was injected i.v. 24 h before. In the upper right quadrant of the NK1.1/CD122 plots, the proportions of CD122+ cells within NK1.1+ CD3– cells (i.e., NK cells) and CD8+ CD3+ cells (i.e., CD8+ T cells), respectively, are given. The proportion of CD122+ cells bound by the i.v. injected CF680-labeled IL-2c is given in the CF680/CD122 plots. **e** Proportion of IL-2-secreting CD45+ cells in spleen, lymph nodes, and tumor. **f** Demonstration that pretreatment with IL-2 strongly reduces the binding of IL-2c to CD122+ cells. Splenocytes were collected from naïve mice. In the blocking setting, CD8+ splenocytes were incubated with IL-2 or (for control) with interferon-β at 37 °C for 30 min. Thereafter, the binding of IL-2c to CD122-positive T cells was determined by flow cytometry

hRT increases the numbers of TILs [31], and up to 30% of the total TIL population of the irradiated tumor was CD122+ (Fig. 5a). We therefore wanted to find out whether the IL-2c tracer could specifically detect TILs in irradiated mice.

Five days after local tumor irradiation with two fractions of 12 Gy, tumor-bearing mice were injected with ^64^Cu-NOTA-IL-2c and imaged 24 h thereafter. As shown in Fig. 5b and Additional file 3, the tracer signal was detectable at the tumor site; however, the tumor uptake was usually only moderate, and in none of the experiments could we detect statistically significant differences between non-depleted mice and CD122-depleted mice (Fig. 5c), although CD122+ TILs were usually successfully depleted in mice injected with the CD122-depleting antibody (see Fig. 5a).

To confirm the paucity of binding to CD122+ cells in the tumor, we determined in different compartments (blood, spleen, tumor) the proportion of CD122+ CD8+ T cells and NK cells that bind to fluorescently labeled IL-2c. As shown in Fig. 5d, 24 h after i.v. injection, substantial binding of fluorescently labeled IL-2c to CD122+ NK cells and CD8+ T cells could be detected in blood and spleen. However, binding in the tumor was much lower (fluorescently labeled IL-2c could only be detected on one-third of the intratumoral CD122+ NK cells and only 3% of the CD122+ CD8+ TILs). These data are in accordance with the PET and ex vivo biodistribution data obtained with radioactively labeled IL-2c (see the high, specific tracer uptake in blood and secondary lymphoid organs in Fig. 4e and f and the moderate, unspecific uptake in the irradiated tumor in Fig. 5b and c).

The lack of strong and specific tracer accumulation in the tumors led us to hypothesize that IL-2 secreted by RT-induced effector cells may compete with therapeutic IL-2c (and the ^64^Cu-NOTA-IL-2c tracer) for binding to CD122/γ_c_ receptors in the tumor microenvironment. Indeed, while only very few leukocytes in secondary lymphoid organs secreted IL-2, many more leukocytes from irradiated tumors secreted it (Fig. 5e). Moreover, an in vitro experiment with CD122+ splenocytes and fluorescently labeled IL-2c showed that pretreatment with IL-2 (but not with an unrelated cytokine) indeed strongly reduces the binding of IL-2c to CD122+ T cells (Fig. 5f). Taken together, our data suggest the possibility that competitive binding of effector T cell-secreted IL-2 to CD122/γ_c_ receptors contributed to the relatively low and unspecific IL-2c tracer uptake in tumors.

### Preferential expansion of extratumoral T and NK cells after treatment with hRT + IL-2c

**Fig. 6:**
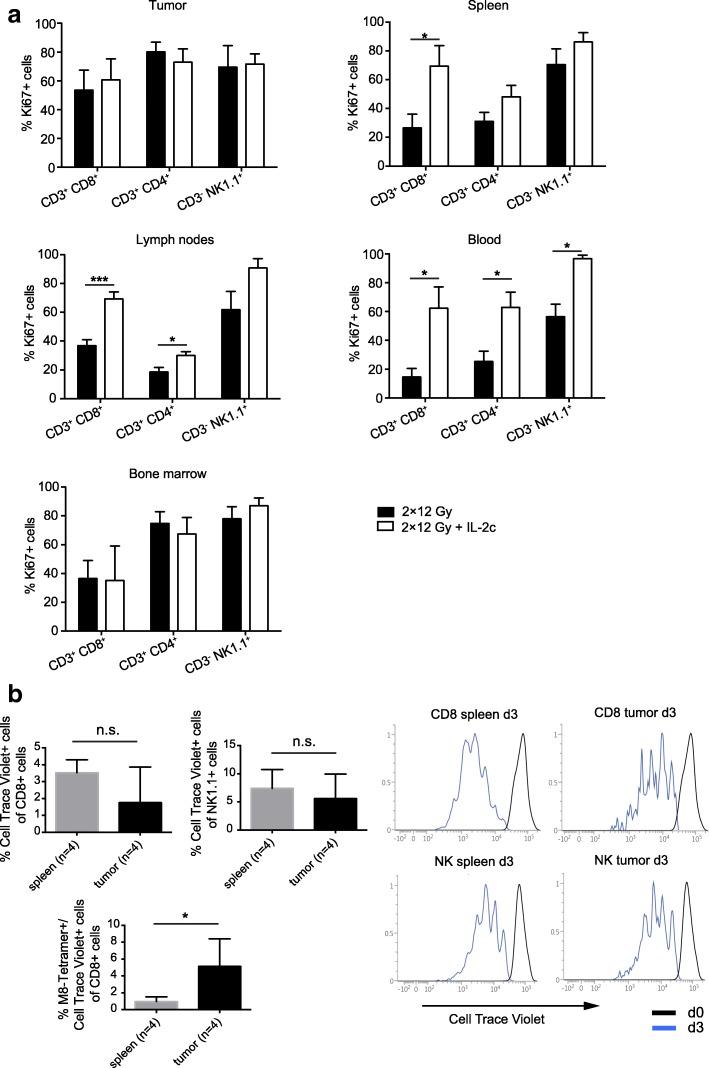
hRT**/**IL-2c combination treatment strongly promotes the expansion of peripheral T and NK cells which can infiltrate the irradiated tumor. **a** Mice bearing a B16-CD133 tumor were treated with hRT alone or hRT followed by i.p. injections of IL-2c for five consecutive days as depicted in Fig. 1b. One day later (corresponding to day 11 after RT), the proportion of proliferating (Ki67+) CD8+ and CD4+ T cells and NK cells from tumor, spleen, lymph nodes, blood, and bone marrow was determined by flow cytometry. **b** CD8+ T and NK cells were isolated from extratumoral compartments (spleen, lymph nodes, and blood) of hRT/IL-2c-treated mice, labeled with Cell Trace Violet and transferred into B16-CD133 tumor-bearing mice 3 days after tumor irradiation without prior lymphodepletion. Daily IL-2c-treatment of the recipient mice was started at the day of the transfer. Three days later, the proportion of donor CD8+ T cells and NK cells (upper left), including tumor-specific, tetramer-binding T cells (lower left), and their proliferation (right) in spleen and tumor of recipient mice were determined by flow cytometry

The findings thus far had shown that i.p. injected IL-2c preferentially bind CD122+ cells in blood and lymphoid tissue and to a much lower extent in the tumor, suggesting that tumor-specific T and NK cells expanded by IL-2c in extratumoral compartments markedly contribute to local tumor control. To substantiate this idea, we analyzed the proliferation of T and NK cells in tumor, spleen, lymph nodes, blood, and bone marrow in hRT- and in hRT/IL-2c-treated mice 11 days after RT. As shown in Fig. 6a, enhanced T and NK cell proliferation – as evidenced by increased expression of the proliferation marker Ki67 – was clearly found in blood, spleen, and lymph nodes but not in irradiated tumor and bone marrow, again suggesting that i.p. injected IL-2c preferentially expand CD122+ cells in blood and secondary lymphoid organs. However, a certain increase in proliferation of tumoral T and NK cells may be difficult to detect because of the already high proportion of proliferating TILs after hRT monotherapy.

To demonstrate that extratumorally expanded T and NK cells can infiltrate the irradiated tumor, we adoptively transferred CD8 T cells and NK cells (6 × 10^6^ and 9 × 10^6^, respectively) pooled from lymph nodes, blood, and spleen of hRT/IL-2c-treated tumor-bearing mice into recipient mice, which were also treated with hRT + IL-2c. As shown in Fig. 6b (upper left), the proportion of adoptively transferred cells in the CD8+ and the NK1.1+ splenocyte populations of the recipient animals was quite low, presumably because the transfer was done without prior lymphodepletion. However, some of the transferred cells infiltrated the irradiated tumor, and, by analyzing the dilution of a cell tracker dye, we found that the infiltrating T and NK cells proliferated in the tumor (Fig. 6b, right). Moreover, tumor antigen-specific T cells were enriched among the transferred TILs (Fig. 6b, lower left), presumably because of stimulation by tumor antigen-presenting cells within the tumor. Taken together, these results suggested that extratumorally expanded T and NK cells can contribute to local control of the irradiated tumor.

### Massive IL-2c-induced expansion of CD122+ lymphocytes is only transient

**Fig. 7:**
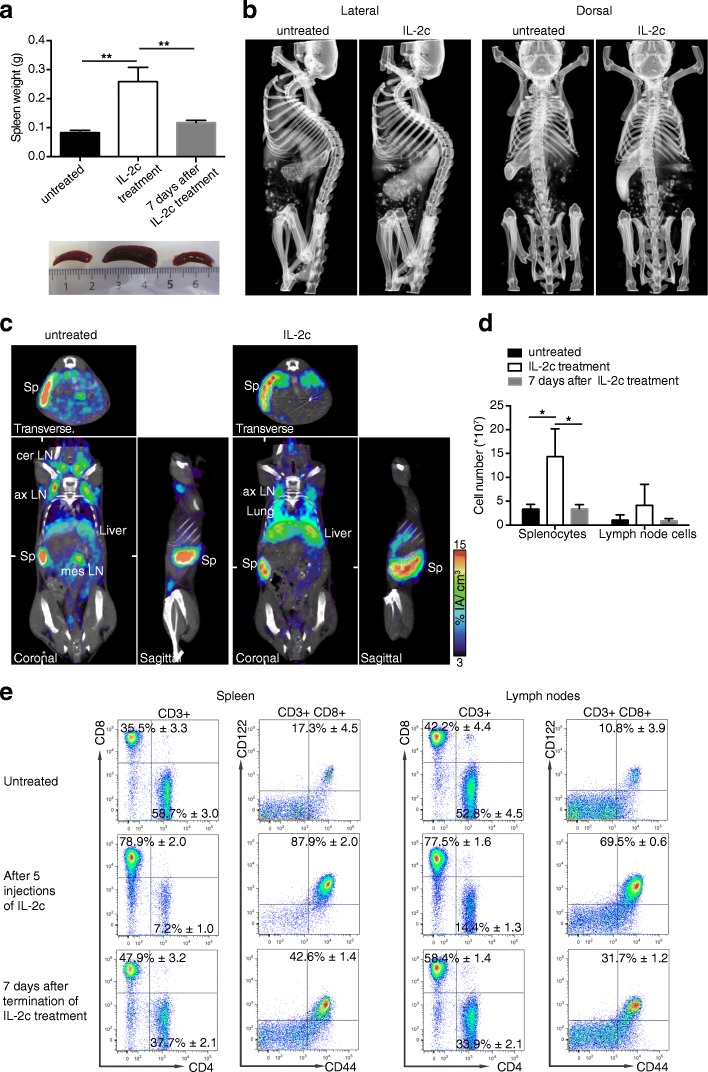
Non-invasive visualization of transient splenomegaly caused by massive, transient expansion of CD122+ lymphocytes. **a** Masses and photographs of representative spleens from untreated mice, mice after a 5-day-long treatment with IL-2c, and 7 days after the last IL-2c injection. **b** Micro-CT images of mice treated with IL-2c for 5 days or not. ExiTron TM 12000 nanoparticles were injected for enhanced spleen contrast. **c** ImmunoPET with ^64^Cu-NOTA-anti-PD-L1. Untreated mice and mice treated for 5 days with IL-2c received 20 μg ^64^Cu-NOTA-anti-PD-L1 (~ 6 MBq); PET images were acquired 24 h later. Representative coronal and transverse PD-L1 immunoPET/CT sections are shown. White ticks in the C-sections indicate the positions of the T- and S-sections. **d** Total cell counts of spleen and lymph nodes. **e** Strong transient expansion of CD122+ CD8+ T cells after treatment with IL-2c as assessed by flow cytometry

IL-2 and CD122/γ_c_-directed IL-2c not only promote the activation and proliferation of antigen-specific T cells but also cause the unspecific expansion of CD8+ T cells and NK cells [13, 15, 16, 34]. Accordingly, five daily injections of IL-2c led to a marked enlargement of the spleen, as shown on photographs of excised spleens and by non-invasive whole-body imaging using contrast-enhanced micro-CT and immunoPET with our previously described ^64^Cu-NOTA-anti-programmed death-ligand 1 (PD-L1) tracer [35] (Fig. 7a-c). Interestingly, this effect turned out to be relatively short-lived. Seven days after cessation of the IL-2c treatment, the spleen sizes had already returned to almost normal ranges (Fig. 7a). The transient nature of this side effect was confirmed by determination of total cell numbers and by flow-cytometric analyses of the CD8+ and CD4+ populations and of CD122^high^ CD44^high^ CD8+ T cells in secondary lymphoid organs (Fig. 7d and e). The PD-L1 immunoPET revealed also an increased PD-L1 tracer uptake in the lungs and the liver after five daily injections of IL-2c, indicating an IL-2c-induced infiltration/expansion of PD-L1-positive leukocytes and/or an upregulation of PD-L1 induced by inflammation in these organs.

## Discussion

We evaluated the antitumor activity of the combination of CD122/γ_c_-directed IL-2c with immunogenic RT. We also describe a PET tracer that allows the non-invasive imaging of IL-2c and of the corresponding bound CD122/γ_c_ receptors. PET imaging and biodistribution studies with this novel tracer prompted experiments that demonstrated that IL-2c do preferentially bind CD122+ NK and CD8+ T cells in blood and secondary lymphoid organs and less efficiently CD122+ cells in the tumor microenvironment. However, the preferential binding to extratumoral CD122+ cells did not preclude effective combination of IL-2c with hypofractionated tumor irradiation.

In fact, the hRT/IL-2c combination had a better antitumor effect on aggressively growing B16 melanoma tumors than hRT alone or hRT + free IL-2. The antitumor effect of the combination depended on CD8+ T cells and to a lesser extent on NK cells and correlated with an increase in tumor-specific T cells both in the tumor and in lymphoid organs. Moreover, cured mice were protected against tumor cell re-challenge, indicating the formation of tumor-specific T cell memory. Synergistic effects of hRT and IL-2c were also observed in the C51 and 4T1 models.

In all three tumor models, IL-2c monotherapy did not affect the growth of the established tumors. This is different to the report by Takahashi et al. ^28^ who, in a highly radiosensitive sarcoma model (with one irradiated and one non-irradiated tumor per mouse), did find growth reduction of the non-irradiated tumor by IL-2c alone, which appears to explain the better overall survival of the mice in their study despite a similar response of the irradiated tumor to RT versus RT + IL-2c. Reduction of tumor growth by CD122-directed IL-2c alone has also been reported by others, but in these studies, treatment usually began early after tumor cell inoculation [7, 13, 14, 36]. Our data from mice with relatively large tumors demonstrate that hRT can greatly enhance the antitumor efficacy of IL-2c. It would be of interest to study this also in orthotopic tumor models in the future.

PET tracers based on uncomplexed IL-2 [37, 38] and a tumor-targeted IL-2 variant [10, 39] have already been developed. The novel PET tracer reported here based on CD122/γ_c_-directed IL-2c allows the non-invasive assessment of the biodistribution of therapeutically active IL-2c and also of their corresponding bound CD122/γ_c_ receptors with high resolution. In naïve mice, the non-invasive imaging of CD122/γ_c_ receptors is particularly difficult as, under normal conditions, only few lymphocytes express this receptor and because these non-activated lymphocytes are small. Nonetheless, we were able to demonstrate specific tracer uptake in secondary lymphoid organs, i.e., in the spleen and to some extent in the lymph nodes, which harbor most CD122+ NK and T cells, and to a lesser extent in the bone marrow.

Although the IL-2c tracer could be detected in B16 melanoma tumors of irradiated mice, the tumor signal turned out to be largely non-specific since it was similar in CD122-depleted mice. Low specific tumor uptake and preferential binding of IL-2c to extratumoral CD122+ cells was confirmed by using fluorescently labeled IL-2c. Preferential binding to extratumoral IL-2 receptor-expressing cells has previously even been demonstrated for an i.v. injected tumor-targeted IL-2 antibody fusion protein and has been explained by the fact that the bispecific fusion protein encountered IL-2 receptor-positive cells earlier (e.g., in the blood) than tumor antigen-positive cells in the tumor [40]. Our data suggest an additional potential mechanism that could contribute to the lack of efficient, specific target binding of IL-2-based therapeutics in tumors. Non-exhausted effector cells in tumors secrete IL-2 [41], and we have shown here that pretreatment with IL-2 blocks the binding of IL-2c to their receptors. Thus, our data suggest the possibility that competition with IL-2 secreted in the tumor microenvironment could also contribute to the low specific tumor targeting of IL-2-based therapeutics. Unspecific tumor uptake of IL-2c may be due to the enhanced pressure and retention effect that causes non-specific tumor trapping of macromolecules [42], as well as due to tumor blood vessels because large amounts of the tracer are circulating in the blood.

By conducting an adoptive transfer experiment with extratumoral T and NK cells from hRT/IL-2c-treated mice, we show that extratumorally expanded T and NK cells, including tumor-specific T cells, infiltrated the irradiated tumor, and therefore extratumorally expanded T and NK cells very likely contributed to local control of the irradiated tumor. This is in line with recent studies suggesting a critical impact of extratumoral responses on tumor rejection in various systems [43, 44]. However, after one injection, we also found fluorescently labeled IL-2c on a small number of tumor-resident NK and CD8+ T cells. Specific binding to tumor-resident lymphocytes may be enhanced after repeated injections or when TILs are more exhausted. Therefore, additional work is necessary to find out to which extent specific tumor uptake of IL-2c is necessary for their antitumor activity. In this respect, it will also be of interest to evaluate the antitumor activity of intratumorally administered IL-2c.

A side effect of IL-2c treatment is the quite dramatic non-specific expansion of CD8+ T and NK cells. This expansion of CD122+ cells was already evident 24 h after the first injection of IL-2c. However, T and NK cell numbers relatively rapidly decreased after treatment cessation. Furthermore, this transient dramatic lymphocyte expansion was not only detected by FACS but also non-invasively. For this purpose, we used contrast-enhanced CT (with a spleen-specific contrast agent) and PD-L1 immunoPET with a theranostic PD-L1 tracer recently developed by us [35]. The PD-L1 tracer is useful for this purpose because most murine leukocytes exhibit some basal PD-L1 expression [35]. The PD-L1 PET not only visualized the splenomegaly but also revealed an enhanced PD-L1 tracer uptake in the liver and the lungs, consistent with IL-2c-mediated expansion of CD122+ cells in these organs. Transient IL-2-mediated inflammation in these organs has previously already been shown by immunohistochemistry for other IL-2-based immunotherapeutics [45]. Liver and lung are typically affected by the IL-2-induced capillary leak syndrome [7, 46]. Thus, the enhanced PD-L1 signal in liver and lung may in part reflect capillary leakage, which, although reduced compared to equally effective doses of free IL-2, can occur following treatment with CD122-directed IL-2c [7, 13]. In any case, imaging approaches that allow the non-invasive detection of T and NK cells and the associated inflammation may be of great value for the further development of IL-2-based drugs [47].

Taken together, we show here that the combination of immunogenic RT with CD122/γ_c_-directed IL-2c can be synergistic and therapeutically more effective than the combination with uncomplexed IL-2 or IL-2c monotherapy. The RT/IL-2c combination could be evaluated in clinical trials with IL-2c directed against human CD122 (such as the recently described NARA1/human IL-2 complexes [13]) and could be an alternative to ICB or RT/ICB combinations, particularly in patients with ICB-induced (auto)immune adverse effects or patients resistant to ICB monotherapy or to combinations with cytotoxic regimens [48]. PET and biodistribution studies with a novel tracer based on IL-2c helped to reveal that IL-2c seem to preferentially bind to and expand CD122+ leukocytes outside of tumor tissue. Similar PET tracers based on IL-2c directed against human CD122/γ_c_ receptors [13] may be of value for the pharmaceutical development of human-specific IL-2c. Further insights into biodistribution kinetics of mouse- or human-specific IL-2c may be obtained with PET tracers labeled with longer-lived radionuclides.

## Additional files

Additional file 1: Description of data: Flow cytometry results showing the successful depletion of CD122-positive cells in spleen and lymph nodes 24 h after i.p. injection of CD122-depleting antibodies. (DOCX 312 kb)

Additional file 2: Title of data: IL-2c immunoPET/CT of naive mice. (MOV 4201 kb)

Additional file 3: IL-2c immunoPET/CT of a tumor-bearing mouse. (MOV 4823 kb)
