# Supplementary material for: Combination treatment with hypofractionated radiotherapy plus IL-2/anti-IL-2 complexes and its theranostic evaluation
Source: J Immunother Cancer. 2019 Feb 26;7:55. doi: 10.1186/s40425-019-0537-9 (PMC6390578; doi:10.1186/s40425-019-0537-9)
Supplement: Supplementary file 1 — Description of data: Flow cytometry results showing the successful depletion of CD122-positive cells in spleen and lymph nodes 24 h after i.p. injection of CD122-depleting antibodies. (DOCX 312 kb) [file 40425_2019_537_MOESM1_ESM.docx]

**Supplementary materials**

**Fig S1**


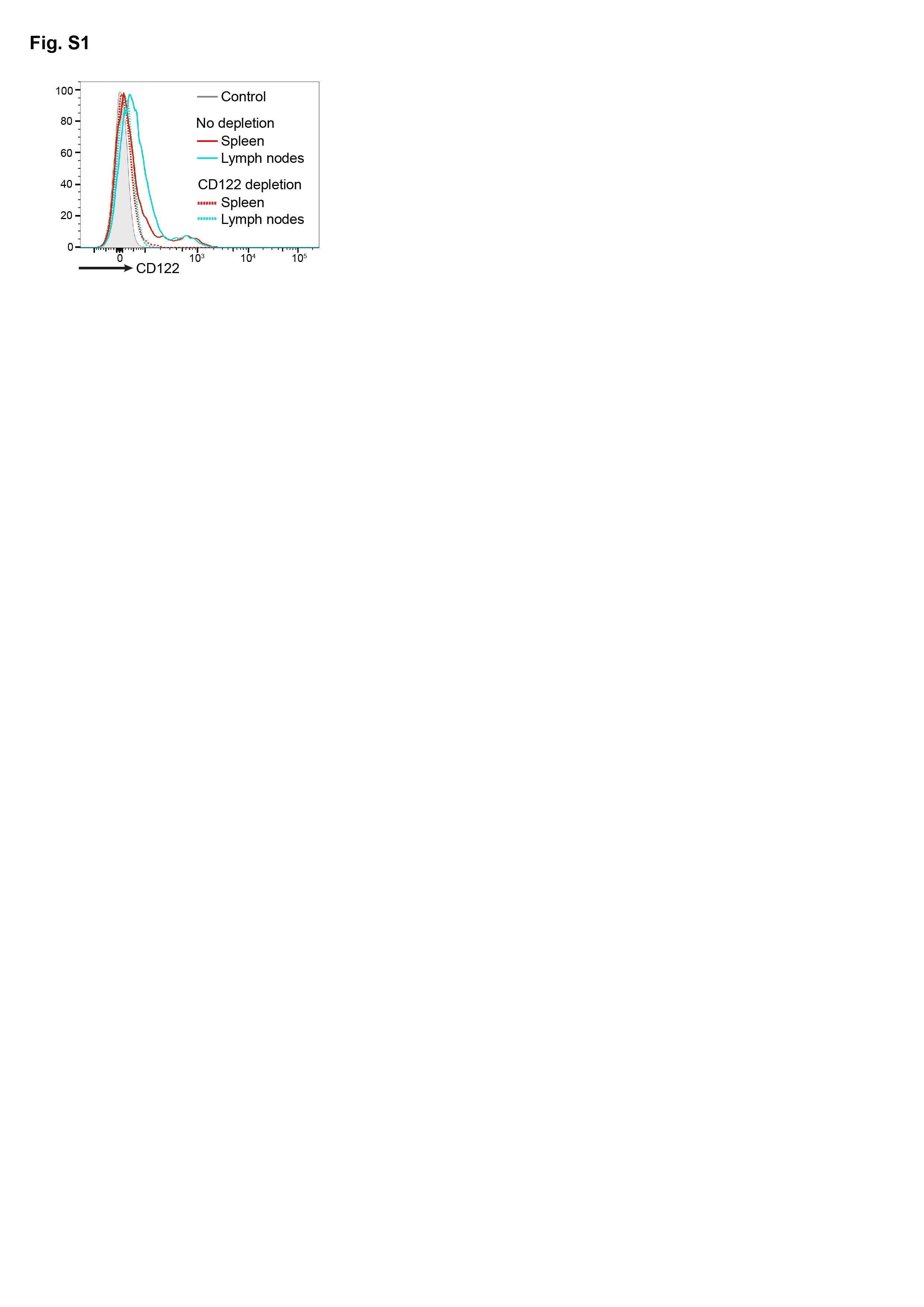


Fig S1. Flow cytometry results showing the successful depletion of CD122-positive cells in spleen and lymph nodes 24 h after i.p. injection of CD122-depleting antibodies.
